# The Asprosin-OLFR734 hormonal signaling axis modulates male fertility

**DOI:** 10.1038/s41421-019-0122-x

**Published:** 2019-11-12

**Authors:** Fangchao Wei, Aijun Long, Yiguo Wang

**Affiliations:** MOE Key Laboratory of Bioinformatics, Tsinghua-Peking Joint Center for Life Sciences, School of Life Sciences, Tsinghua University, Beijing, 100084 China

**Keywords:** Mechanisms of disease, Cell signalling

Dear Editor,

Approximately 15% of couples of childbearing age have fertility problems, and more than a quarter of infertility cases can be attributed to decreased male sperm quality^1^. Sperm quality indicators include sperm count, sperm viability and sperm progressive motility^2^. Male infertility is associated with many factors, including environmental toxins, abuse of alcohol and tobacco, genetics, and accumulated negative emotional responses^1–3^. In addition, obesity and aging are major contributors to human infertility. Obesity has been shown to disturb the hypothalamic-pituitary-gonadal axis, which results in the inability of the gonads to provide physiological levels of testosterone and a normal number of spermatozoa^1–3^. In modern life, more and more couples choose to postpone having a child due to various socioeconomic reasons, but aging causes genetic and epigenetic changes in spermatozoa^1–3^. Semen volume, sperm concentration, sperm progressive mobility and the percentage of morphologically normal sperm all begin to decrease after the age of 40^1–3^.

Olfactory receptors (ORs), which comprise almost half of the GPCR (G protein-coupled receptor) family, belong to the Rhodopsin family, one of five GPCR families. Most ORs are highly expressed in the olfactory epithelium and the olfactory bulbs to sense environmental chemical changes^4^. However, many studies show that some ORs are highly expressed in peripheral tissues^4^. Transcriptome analyses have deciphered all human testicular and spermatozoa OR expression patterns and demonstrated that the testis is the most OR transcript-rich of all tissues, apart from the nose^5^. It was reported that a couple of olfactory receptors modulate sperm chemotaxis^4,6^, but it is still unclear whether and how other olfactory receptors function in sperm motility and/or chemotaxis.

Our previous results show that olfactory receptor OLFR734 is a receptor of Asprosin, a fasting-induced gluconeogenic hormone^7^. OLFR734 is highly expressed in testis evaluated by quantitative PCR (qPCR) (Fig. 1a), but its role in testis is unknown. Compared to wildtype (WT) mice, 10-week-old male *Olfr734*^−/−^ mice have similar body weight, blood glucose level, testis weight, testis morphology and testis histology (Supplementary Fig. S1a–S1e). To further evaluate the possible role of OLFR734 in testis function, we measured sperm quality. As shown in Supplementary Fig. S1f–S1h, knockout of *Olfr734* has no effect on sperm number, sperm viability and sperm morphology, indicating that spermiogenesis is normal in *Olfr734*^−/−^ mice. However, progressive motility—the ability of sperm to move straight forward in a clearly defined direction—was severely diminished in *Olfr734*^−/−^ sperm (Fig. 1b). Consistent with this result, the percentage of sperm with slow motility was dramatically increased in *Olfr734*^−/−^ mice (Fig. 1b). Since progressive motility is essential for spermatozoon movement in the female reproductive tract, we measured fertility by mating male WT or *Olfr734*^−/−^ mice with female WT mice. The fertilization potential of *Olfr734*^*−/−*^ mice was significantly diminished (Fig. 1c). In addition, the plasma testosterone levels and frequency of copulation plugs were not affected in *Olfr734*^−/−^ mice (Supplementary Fig. S1i and S1j), which indicates that the OLFR734-mediated pathway in fertility is different to the testosterone-mediated one and the mating behavior is similar in WT and *Olfr734*^*−/−*^ mice. The similar *in vitro* fertilization results (Supplementary Fig. S1k) further suggest that the decreased fertility in *Olfr734*^−/−^ mice is determined by a sperm-specific effect in vivo (for example, on motility) of *Olfr734* deficiency. Sperm must be highly motile for a long time over a considerable distance to fertilize with egg, and they must contain high levels of ATP^8^. The lower progressive motility and fertilization potential suggest that sperm ATP levels are attenuated. In support of this notion, the ATP content of *Olfr734*^−/−^ sperm was obviously decreased (Fig. 1d). Taken together, these results demonstrate that OLFR734 promotes sperm progressive motility and enhances male fertility.

**Fig. 1:**
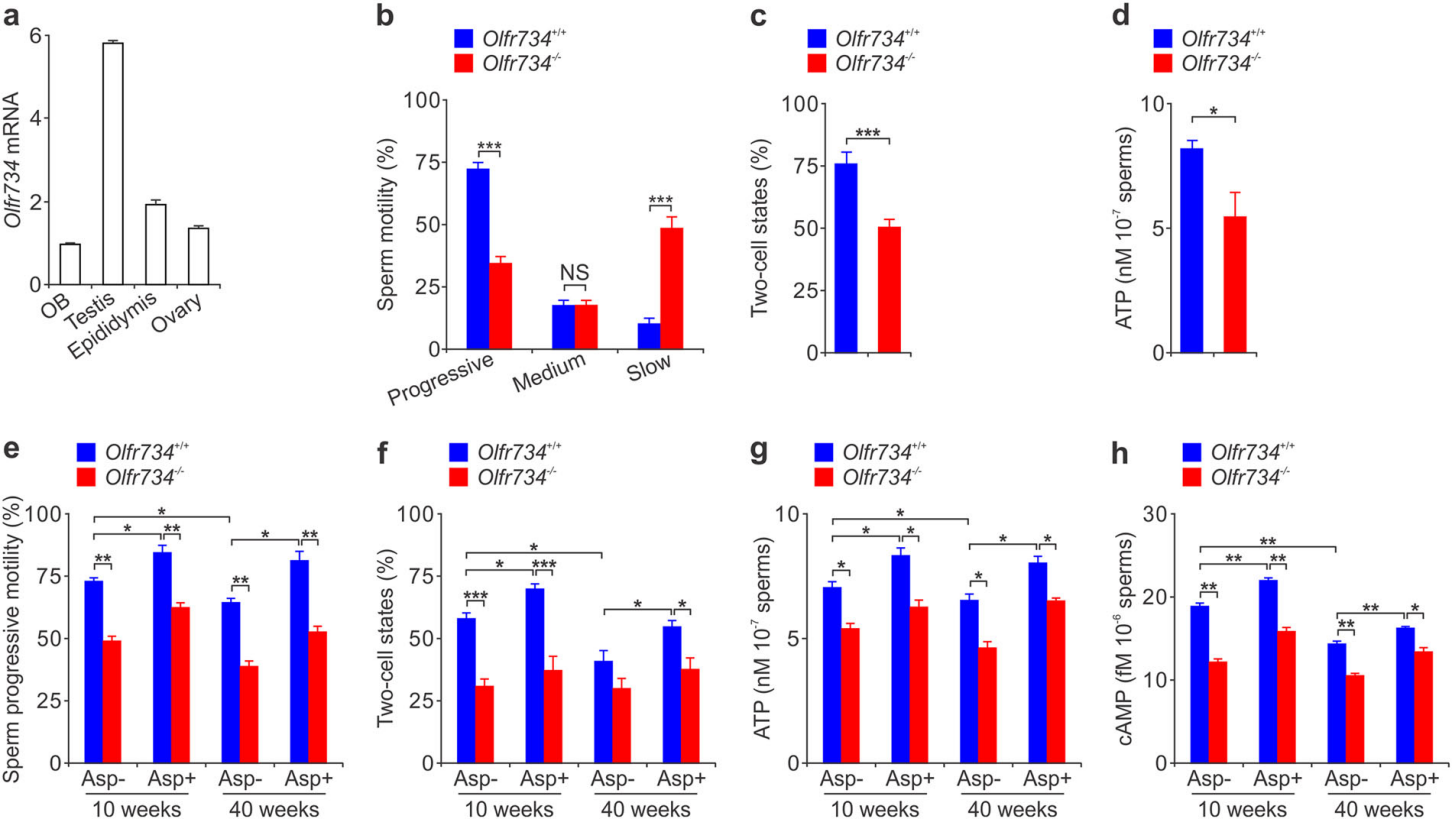
The Asprosin-OLFR734 signaling axis promotes sperm progressive motility and enhances male fertility. **a** qPCR results showing relative mRNA levels of *Olfr734* in different tissues from mice. Data are shown as mean ± s.e.m. *n* = 5 mice. **b–d** Sperm motility (**b**), two-cell states (**c**) and sperm ATP content (**d**) from 10-week-old WT and *Olfr734*^−/−^ *ad lib*-fed mice. To calculate two-cell states, 10 WT female mice were mated to 5 male WT or *Olfr734*^−/−^ mice (two female mice for one male mouse). *NS*, no significant statistical difference. Data are shown as mean ± s.e.m. *P < 0.05, ***P < 0.001, *n* = 5 mice. **e**, **f** Sperm progressive motility (**e**) and two-cell states (**f**) from 10-week-old or 40-week-old WT and *Olfr734*^−/−^ mice. Mice were intraperitoneally injected with purified GST or GST-Asprosin (60 μg kg^-1^) for 10 days before isolation of sperm. To calculate two-cell states, 10 WT female mice were mated to 5 male WT or *Olfr734*^−/−^ mice (two female mice for one male mouse). Data are shown as mean ± s.e.m. *P < 0.05, **P < 0.01, ***P < 0.001, *n* = 5 mice. **g**, **h** ATP (**g**) and cAMP (**h**) content in sperm from 10-week-old or 40-week-old WT and *Olfr734*^−/−^ mice. Mice were intraperitoneally injected with purified GST or GST-Asprosin (60 μg kg^-1^) for 10 days before isolation of sperm. Data are shown as mean ± s.e.m. *P < 0.05, **P < 0.01, *n* = 5 mice

cAMP signaling is critical for sperm capacitation, motility, and the acrosome reaction, and Asprosin binds OLFR734 and activates cAMP signaling^7,9,10^. Since OLFR734 promotes sperm progressive motility and enhances fertility, we hypothesized that Asprosin treatment should have a similar effect. To confirm this hypothesis, we purified active Asprosin (Supplementary Fig. S2a and S2b) and then tested the effect of the Asprosin-OLFR734 signaling axis on sperm physiology. Although Asprosin administration increased blood glucose in WT mice but not in *Olfr734*^−/−^ mice, the Asprosin-OLFR734 signaling axis had no effect on body weight, testis weight, testis morphology, testis histology, sperm number, sperm viability, and sperm morphology (Supplementary Fig. S2c–S2j). Similar conclusions were obtained from 10-week-old or 40-week-old WT and *Olfr734*^−/−^ mice (Supplementary Fig. S2c–S2j). Asprosin treatment enhanced sperm progressive motility in 10-week-old mice, while *Olfr734* deficiency almost abolished this effect (Fig. 1e). Similar results were observed in sperm from 40-week-old mice. In addition, sperm progressive motility in 40-week-old mice was much lower than that in 10-week-old mice (Fig. 1e). Since the Asprosin-OLFR734 axis is critical for sperm progressive motility, we next tested the effect of this axis on fertilization potential. Asprosin increased the fertilization ratio in both 10-week-old and 40-week-old WT mice but not in *Olfr734*^−/−^ mice (Fig. 1f). In support of this, the ATP and cAMP levels in sperm were dramatically enhanced by Asprosin treatment (Fig. 1g, h). More importantly, Asprosin treatment almost restored the ATP and cAMP content, sperm progressive motility and fertilization potential in 40-week-old mice to levels that were comparable to 10-week-old mice (Fig. 1e–h). In addition, the Asprosin-OLFR734 signaling axis has a protective role against high fat diet-induced deterioration of sperm motility (Supplementary Fig. S3). Together, these results indicate that the Asprosin-OLFR734 axis promotes sperm progressive motility and male fertility.

Asprosin acts as a fasting-induced hormone and binds OLFR734 to activate cAMP signaling and promote hepatic glucose production^7,10^. In this study, we demonstrate that the Asprosin-OLFR734 signaling axis promotes sperm progressive motility and enhances fertility. In addition, Asprosin treatment can restore sperm progressive motility in old mice to an extent comparable to that in young mice, which suggests that Asprosin or agonists of OLFR734 are potential drugs to improve fertility. Of note, activation of OLFR734 signaling may increase the risk of hyperglycemia. Thus, it is important to find the cues that specifically activate OLFR734 in the testis.

ATP is mainly derived from glycolysis and oxidative phosphorylation. Previous reports show that both glycolysis and oxidative phosphorylation are critical for sperm motility^8^. In the future, it will be important to determine the glycogen and glucose levels, as well as the critical steps of ATP production, to understand the exact mechanism of Asprosin-OLFR734 on sperm physiology.

Previous reports show that some olfactory receptors promote sperm chemotaxis^6^. It is unclear whether OLFR734 also modulates sperm chemotaxis. Since Asprosin is an endocrine factor, it cannot directly modulate sperm chemotaxis. However, OLFR734, as an olfactory receptor, may respond to certain odorants to regulate sperm chemotaxis. Therefore, it is critical to determine the corresponding odorants. In addition, the mechanism that coordinates these odorants with Asprosin deserves further investigation.

## Supplementary information

Supplementary information

## Supplementary information

**Supplementary Information** accompanies the paper at (10.1038/s41421-019-0122-x).
