# Author Correction: The Asprosin-OLFR734 hormonal signaling axis modulates male fertility

**DOI:** 10.1038/s41421-025-00834-9

**Published:** 2025-09-19

**Authors:** Fangchao Wei, Aijun Long, Yiguo Wang

**Affiliations:** MOE Key Laboratory of Bioinformatics, Tsinghua-Peking Joint Center for Life Sciences, School of Life Sciences, Tsinghua University, Beijing, 100084 China

Correction to: *Cell Discovery* 10.1038/s41421-019-0122-x, published online 12 November 2019

In the originally published version of this article, the image labeled “Hematoxylin and eosin staining of testis from 10-week-old *Olfr734*^−/−^ mice intraperitoneally injected with GST-Asprosin” in Supplementary Fig. S2g was misplaced. This error does not affect the conclusions of the paper.

## Supplementary information

new supplementary file

