# Supplementary material for: Author Correction: The Asprosin-OLFR734 hormonal signaling axis modulates male fertility
Source: Cell Discov. 2025 Sep 19;11:78. doi: 10.1038/s41421-025-00834-9 (PMC12449444; doi:10.1038/s41421-025-00834-9)
Supplement: Supplementary file 1 — new supplementary file [file 41421_2025_834_MOESM1_ESM.pdf]

Materials and methods

Mice

Mice were housed in a temperature-controlled environment using a 12 hr light/12 hr dark cycle. Animals had *ad libitum* access to water at all times. *Olfr734*$^{-/-}$ mice were generated as previously reported[1]. Genotypes were confirmed by PCR using the following primers: 5'-ACCTGCAAATGATACCACCGT-3' and 5'-TCCGGACAACCTGAGTGATG-3'. All mice were maintained on a C57BL/6J background. All procedures were approved by the Animal Care and Use Committee at Tsinghua University.

Cell culture

Mouse primary hepatocytes were isolated as previously described[1] and cultured in M199 medium containing 2% FBS and 0.2% BSA until attached, then continuously cultured in M199 medium without FBS. To evaluate the activity of purified GST-Asprosin, primary hepatocytes were treated with 100 nM GST or GST-Asprosin for 2 hrs.

Sperm separation and motility analysis

Epididymal spermatozoa from adult male mice were collected by swim-out from caudal epididymis in M2 medium (Sigma, M7167). Sperm were placed in a slide chamber and motility was examined using a Hamilton Thorne IVOS II Sperm Analyzer with a stage temperature of 37°C. Sperm motility (%) was quantified and motion parameters were measured.

Fertilization test

Four-week-old C57BL/6J female mice were super-ovulated by injecting progesterone and anti-inhibin serum. Ten female mice were caged with 5 *Olfr734*$^{+/+}$ or *Olfr734*$^{-/-}$ males (2 females per male) for two days, to record pregnancy and 2-cell embryos.

Measurement of cellular ATP, cAMP, blood glucose levels and plasma testosterone levels

ATP levels and cAMP levels were measured in sperm lysates using the ATP determination kit (Thermo Fisher, A22066) and the Cyclic AMP ELISA Kit (581001, Cayman), respectively, according to the manufacturer’s instructions. Blood glucose values were determined using a LifeScan automatic glucometer. Plasma testosterone levels were measured using a Testosterone ELISA Kit (Beyotime, PT872) according to the manufacturer’s instructions.

Recombinant protein purification

Mouse *Asprosin* was cloned into a pGEX-4T-1 vector for expression in *E. coli*. Bacterially expressed GST and GST-Asprosin were purified as previously reported[1]. The purified proteins were subjected to SDS-PAGE analysis in order to determine protein purity. The GST and GST-Asprosin proteins were >90% pure with endotoxin concentration equal to or below 2 EU ml^{-1}.

Quantitative PCR

Total RNA from cells or mouse tissues was extracted using a Total RNA Purification kit (Omega, R6812-02). cDNA was obtained using the RevertAid First Strand cDNA Synthesis kit (Thermo, K1622). RNA levels were measured

with the LightCycler 480 II (Roche) as previously described[1]. The following primers were used for qPCR:
Actin-Forward: 5’-GTCCACCCCGGGGAAGGTGA-3’
Actin-Reverse: 5’-AGGCCTCAGACCTGGGCCATT-3’
G6pc-forward: 5'-GTGAATTACCAAGACTCCCAGGACTG-3'
G6pc-reverse: 5'-GATGGAACCAGATGGGAAAGAGGAC-3'
Olfr734-forward: 5'-CTCAGACTCGGGAAGTGCAG-3'
Olfr734-reverse: 5'-AAGCATCTTAGGCGCTGTGA-3'

Statistical analyses

Mice were randomly assigned for the experiments. The number of animals used in each experiment is outlined in the corresponding figure legends. No animals were excluded from statistical analyses, and the investigators were not blinded in the studies. All studies were performed on at least three independent occasions. Results are reported as mean ± S.E.M. Comparison of different groups was carried out using two-tailed unpaired Student’s *t*-test or one-way ANOVA. Differences were considered statistically significant at $P < 0.05$.

Reference

1 Li, E. *et al.* OLFR734 Mediates Glucose Metabolism as a Receptor of Asprosin. *Cell Metab* **30**, 319-328 e318, doi:10.1016/j.cmet.2019.05.022 (2019).

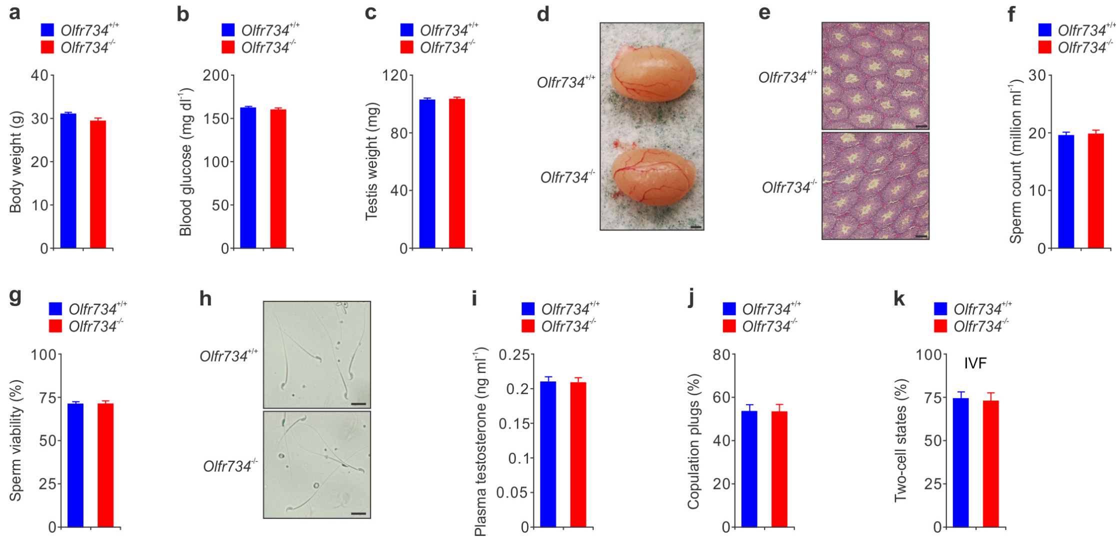

Supplementary Figure S1: Effect of *Olfr734* deficiency on fertilization.
a-b Body weight (**a**) and blood glucose levels (**b**) from 10-week-old WT and *Olfr734*$^{-/-}$ *ad lib*-fed mice. Data are shown as mean ± s.e.m. n = 5 mice. **c-d** Testis weight (**c**) and morphology (**d**) from 10-week-old WT and *Olfr734*$^{-/-}$ *ad lib*-fed mice. Scale bar, 1 mm. Data are shown as mean ± s.e.m. n = 5 mice. **e** Hematoxylin and eosin staining of testis from 10-week-old WT and *Olfr734*$^{-/-}$ *ad lib*-fed mice. Scale bars, 100 μm. **f-h** Sperm count (**f**), sperm viability (**g**) and sperm morphology (**h**) from 10-week-old WT and *Olfr734*$^{-/-}$ *ad lib*-fed mice. Scale bars, 50 μm. Data are shown as mean ± s.e.m. n = 5 mice. **i** Plasma testosterone levels from 10-week-old WT and *Olfr734*$^{-/-}$ *ad lib*-fed mice. Data are shown as mean ± s.e.m. n = 5 mice. **j** Frequency of copulation plugs (percentage of pairings after which a plug was observed). 24 WT female mice were mated to 12 male WT or *Olfr734*$^{-/-}$ mice (two female mice for one male mouse) and copulation plugs were counted. Data are shown as mean ± s.e.m. **k** Two-cell states following *in vitro* fertilization by sperm from 10-week-old WT and *Olfr734*$^{-/-}$ *ad lib*-fed mice. Data are shown as mean ± s.e.m. n = 5 mice.

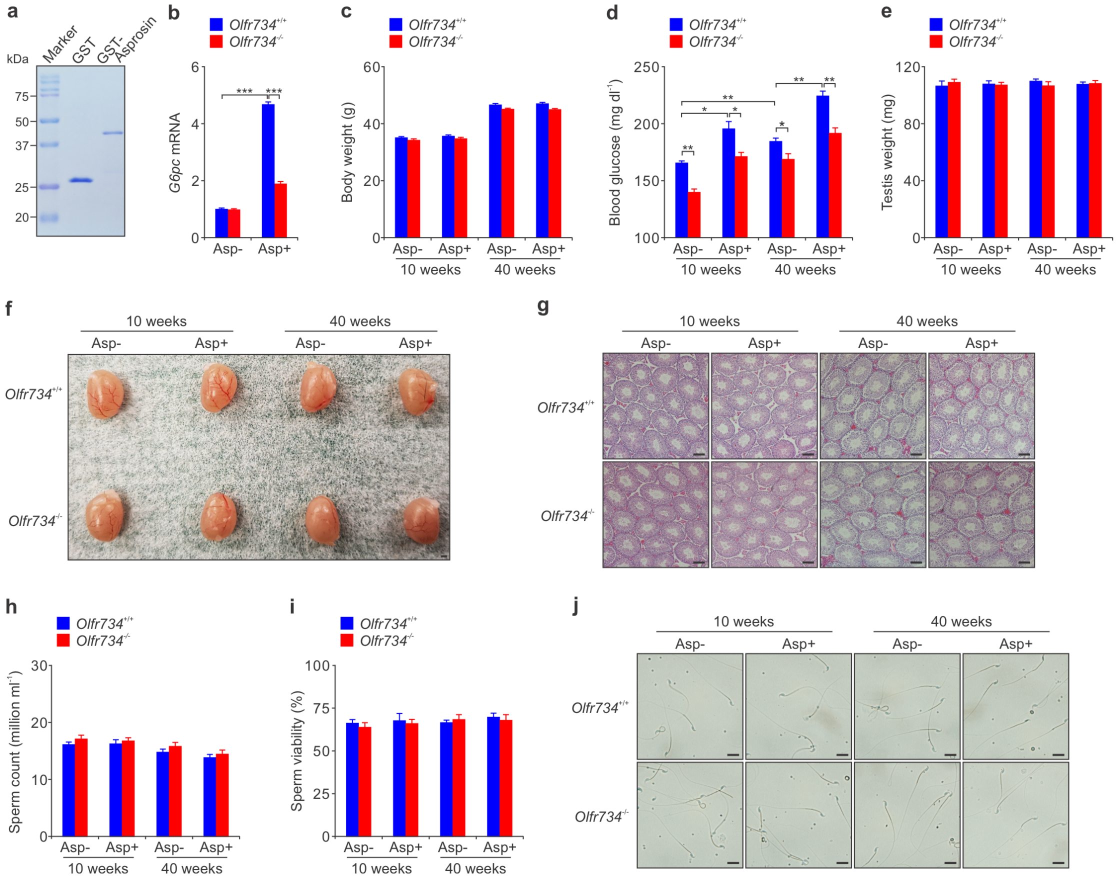

Supplementary Figure S2: Effect of Asprosin on fertilization.

a Coomassie staining showing GST and GST-Asprosin after purification from *E. coli.* **b** qPCR results showing the effect of purified GST and GST-Asprosin on *G6pc* expression. Data are shown as mean ± s.e.m. ****P* < 0.001, n = 6. **c-d** Body weight (**c**) and blood glucose levels (**d**) from 10-week-old or 40-week-old WT and *Olfr734*$^{-/-}$ mice. Mice were intraperitoneally injected with purified GST or GST-Asprosin (60 μg kg^{-1}) for 10 days before testing. Data are shown as mean ± s.e.m. **P* < 0.05, ***P* < 0.01, n = 5 mice. **e-f** Testis weight (**e**) and morphology (**f**) from 10-week-old or 40-week-old WT and *Olfr734*$^{-/-}$ mice. Mice were intraperitoneally injected with purified GST or GST-Asprosin (60 μg kg^{-1}) for 10 days before testing. Scale bar, 1 mm. **g** Hematoxylin and eosin staining of testis from 10-week-old or 40-week-old WT and *Olfr734*$^{-/-}$ mice. Mice were intraperitoneally injected with purified GST or GST-Asprosin (60 μg kg^{-1}) for 10 days before testing. Scale bars, 100 μm. **h-j** Sperm number (**h**), sperm viability (**i**) and sperm morphology (**j**) from 10-week-old or 40-week-old WT and *Olfr734*$^{-/-}$ mice. Mice were intraperitoneally injected with purified GST or GST-Asprosin (60 μg kg^{-1}) for 10 days before isolation of sperm. Scale bars, 50 μm. Data are shown as mean ± s.e.m. n = 5 mice.

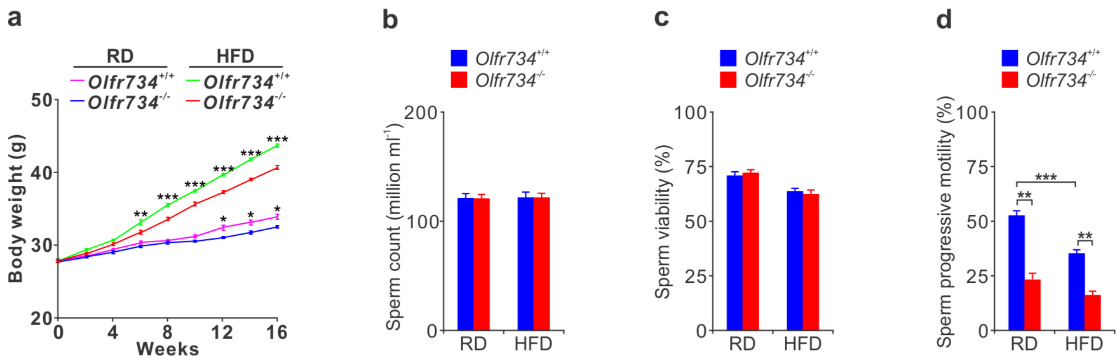

Supplementary Figure S3: Effect of high fat diet-induced obesity on fertilization.

a-d Body weight curves (**a**), sperm number (**b**), sperm viability (**c**) and sperm progressive motility (**d**) from WT and *Olfr734*$^{-/-}$ mice fed on a RD or HFD for 8 weeks. Data are shown as mean ± s.e.m. **P* < 0.05, ***P* < 0.01, ****P* < 0.001, n = 5 mice.
